# Cross-sectional Associations of Self-Perceptions of Aging With Self-Efficacy, Depressive Symptoms, and Satisfaction With Life in Dementia Caregivers and Non-Caregivers

**DOI:** 10.1177/00914150251382810

**Published:** 2025-10-09

**Authors:** Serena Sabatini, Shelbie Turner

**Affiliations:** 1 School of Psychology, Faculty of Health and Medical Sciences, 3660 University of Surrey, Guildford, UK; 2 Department of Clinical Psychology and Psychobiology, University of Barcelona, Barcelona, Spain; 3 Division of Geriatrics and Palliative Medicine, Weill Cornell Medical College, New York City, USA

**Keywords:** carers, Alzheimer's, subjective aging, felt age, attitudes towards own aging, age-related cognitions, self-perceptions

## Abstract

This study estimated the cross-sectional associations of self-perceptions of aging with self-efficacy, depressive symptoms, and life satisfaction in dementia caregivers and non-caregivers; and tested whether these associations are stronger among dementia caregivers compared to non-caregivers. Data from the German Aging Study comprising 190 dementia caregivers (mean age = 65.69 years; SD = 10.11) and 4480 non-caregivers (mean age = 68.81 years; SD = 10.49) was used. Felt age, attitudes towards own aging, age-related cognitions, self-efficacy, depressive symptoms, and life satisfaction were assessed. Regression models were estimated. Association for younger felt age with greater self-efficacy; younger felt age, more positive attitudes towards own aging, and fewer perceived physical losses with fewer depressive symptoms; and younger felt age and more positive attitudes towards own aging with greater life satisfaction were stronger for dementia caregivers than non-caregivers. Positive self-perceptions of aging may help maintaining self-efficacy, good mood, and life satisfaction when assuming challenging roles such as caregiving.

Self-perceptions of aging refer to peoples’ appraisals of their own aging. Over the past three decades several concepts and measures assessing self-perceptions of aging have been developed. Examples are felt age, attitudes towards own aging, and age-related cognitions (AgeCog). Felt age assesses how old people feel they are relative to their chronological age (Barrett, 2003). A younger felt age generally indicates positive self-perceptions of aging whereas an older felt age indicates negative self-perceptions of aging, attitudes towards own aging comprises an explicit and overall personal evaluation of age-related changes (Lawton, 1975). AgeCog assess age-related cognitions in different domains such as physical losses, social losses, and continuous growth and ongoing development (Steverink et al., 2001).

Cross-sectional and longitudinal evidence has repeatedly shown that middle-aged and older adults with more positive self-perceptions of aging benefit from better physical, mental, and cognitive health, greater wellbeing, and greater satisfaction with life (Mirucka et al., 2016; Montepare & Lachman, 1989; Sabatini et al., 2020; Tully-Wilson et al., 2021; Westerhof et al., 2023). More positive self-perceptions of aging are also associated with less presence of indicators of physiological aging (e.g., telomere length; markers of inflammation) (Sabatini et al., 2025). According to Levy's Stereotype Embodiment Theory (Levy, 2009) and related empirical evidence, the connection between more positive self-perceptions of aging and better health outcomes exists because people with more positive self-perceptions of aging possess more adaptive psychological characteristics (e.g., internal control; greater self-efficacy) that, in turn, foster engagement with health-enhancing behaviors (e.g., physical exercise; having a balanced diet) (Tovel et al., 2019; Turner & Hooker, 2022). Levy and colleagues estimated that U.S. nation-wide healthcare expenditure due to negative self-perceptions of aging each year is $33.7 billion (Levy et al., 2020).

Self-perceptions of aging are not only useful to promote active and healthy aging, but may also foster greater adaptation to age-related limitations and health issues (Dutt et al., 2016). For example, among community-dwelling people with dementia more positive self-perceptions of aging were found related to greater emotional wellbeing and quality of life (Sabatini et al., 2021, 2024a). Among people with a diagnosis of cancer more positive self-perceptions of aging were associated with less perceived disability, greater satisfaction with recovery, greater self-efficacy beliefs, and more use of adaptive coping strategies (Boehmer, 2007). Importantly, self-perceptions of aging are malleable to change. Hence, health and adaptation to age-related challenges can be fostered though existing psychoeducational and behavioral interventions promoting positive self-perceptions of aging (Gilhooly et al., 2016; Knight et al., 2021).

Positive self-perceptions of aging may be a particularly useful resource that helps prevent negative caregiving-related outcomes in informal dementia caregivers (i.e., family and friends helping and supporting the person with dementia) (Sabatini et al., 2024a, 2024b), and caregiving itself might contribute to a person's self-perceptions of aging. Global estimations suggest there are 55 million people with dementia (Alzheimer's Disease International, 2023). With the progression of dementia, individuals’ cognitive and functional difficulties become more severe and, because of this, people with dementia become increasingly dependent on others. About 60% of people with dementia resides in the community and are supported by informal caregivers (Alzheimer's Society, 2014). The number of informal dementia caregivers in the UK is around 670,000 (Alzheimer's Society, 2024) and in the US it is over 16 million (Centers for Disease Control and Prevention).

Using a large sample of German middle-aged and older individuals, the current study aims to estimate the associations of self-perceptions of aging with self-efficacy, satisfaction with life, and depressive symptoms in dementia caregivers and non-caregivers, as well as to investigate whether the size of these associations differ between dementia caregivers and non-caregivers. The focus on self-efficacy, satisfaction with life, and depressive symptoms is due to these variables being important outcomes for the wellbeing of caregivers and the person they care for (Gallagher et al., 2011; Lamont et al., 2019; McDermott et al., 2014). Self-efficacy captures a person's belief in their capacity to control aspects of their lives and complete given tasks (Schwarzer & Jerusalem, 1995). Examples of depressive symptoms are low mood, changes in one's sleep and appetite, decreased motivation and lack of interest in things (American Psychiatric Association, 2013). Satisfaction with life consists in the feelings and attitudes that an individual has about their own life and at a particular point of their life (Diener et al., 1985).

Prior evidence on self-perceptions of aging and caregiving outcomes among dementia caregivers is limited. First, more positive attitudes towards own aging were found significantly associated with greater self-efficacy both in Kim and Bolkan (2022) and in Sabatini et al. (2024a). Second, more positive attitudes towards own aging has been related to fewer depressive symptoms in Australian caregivers of older people in general (Loi et al., 2015), but self-perceptions of aging have never been empirically related to depressive symptoms in dementia caregivers. However, more positive attitudes towards own aging have been found related to other indicators of mood (i.e., more positive and less negative emotions) in dementia caregivers (Kim & Bolkan, 2022). Third, among UK dementia caregivers, a younger felt age (indicating positive self-perceptions of aging) was associated with greater satisfaction with life (Sabatini et al., 2024b). Overall, self-perceptions of aging may be associated with greater self-efficacy, higher satisfaction with life, and fewer depressive symptoms in dementia caregivers, but more evidence is needed.

Knowing whether the strength of the associations of self-perceptions of aging with self-efficacy, satisfaction with life, and depressive symptoms differ between dementia caregivers and non-caregivers can offer insight into caregiving's role in people's appraisal of their own aging, as well as how those appraisals impact health and well-being outcomes. But, this question has never been tested. It may be that the adaptive function of positive self-perceptions of aging is stronger and has a bigger effect on self-efficacy, satisfaction with life, and mood of those individuals facing challenging roles such as caregiving. Indeed, caregiving for a person with dementia may give rise to new depressive symptoms or exacerbate the depressive symptoms a person may have had (Collins & Kishita, 2020; Mahoney et al., 2005). Positive self-perceptions of aging may be even more important in these situations to buffer the negative effects of caregiving on caregiving-related outcomes. Hence, we hypothesize that the cross-sectional associations of self-perceptions of aging with self-efficacy, satisfaction with life, and depressive symptoms are stronger in dementia caregivers compared to non-caregivers.

## Methods

### Description of German Ageing Survey

This study uses cross-sectional data from the German Ageing Survey (Klaus et al., 2017), which is a nationwide representative cohort-sequential survey of the German community-dwelling population aged  ≥ 40 years. The German Ageing Survey started in 1996 and is currently ongoing. Since the beginning of the German Ageing Survey, every six years a new baseline sample has been recruited. This study uses data collected through self-administered questionnaires during the most recent baseline sample of 2021.

### Measures

**Felt age** was assessed with a single-item question asking participants to write in years the age that they feel most of the time. A proportional discrepancy score was calculated by subtracting participants’ felt age from their chronological age, and by dividing this difference score by participants’ chronological age. Positive values indicate youthful felt age, whereas negative values indicate older felt age. In the study analyses felt age was used as a continuous variable.

**Attitudes towards own aging** were assessed with the five-item attitudes towards own aging scale, taken from the Philadelphia Geriatric Center Morale Scale (Lawton, 1975). For each item respondents have to make temporal comparisons about changes in energy level, perceived usefulness, happiness, and quality of life. Answer options are binary and vary based on the framing of the item (i.e., better versus worse, yes versus no). A sample item is “*Things keep getting worse as I get older.”* A proportion-based score was obtained by summing the participant's item scores and by dividing it by the number of completed responses. Attitudes towards own aging were used as a continuous variable with a score of one indicating positive attitudes in all answers and a score of zero indicating negative attitudes in all answers. In this study sample, Cronbach's alpha was 0.78, indicating excellent internal reliability.

**Age-related cognitions** were assessed with three of the four AgeCog scales assessing physical losses; social losses; and continuous growth and ongoing development (Steverink et al., 2001). Each scale comprises four items. Each item starts with the stem “*Aging means to me that….”* A sample item assessing physical losses is: “*…I am less energetic and fit.”* A sample item assessing social losses is: “*…I feel less needed.”* A sample item assessing continuous growth and ongoing development is: “*…I can still learn new things.*” Participants indicate the extent to which each item applies to them using a four-point scale ranging from *Strongly disagree* to *strongly agree*. Higher scores indicate more negative AgeCog in the physical losses and social losses scales but more positive AgeCog in the continuous growth and ongoing development scale. Cronbach's alpha for the physical losses scale was 0.79 (excellent internal reliability), for the social losses scale was 0.73 (good internal reliability), for the continuous growth and ongoing development scale was 0.80 (excellent internal reliability). The self-knowledge scale was not used in this study due to lower reliability in this study sample (i.e., Cronbach's alpha of 0.62).

**Self-efficacy.** We measured self-efficacy via the five-item Generalized Self-Efficacy Scale (Schwarzer & Jerusalem, 1995). Example items are “*It is easy for me to stick to my aims and accomplish my goals*” and “*I can usually handle whatever comes my way.”* For each item participants have to choose an answer option on a four-point scale (1 = Strongly agree; 2 = Agree; 3 = Disagree; 4 = Strongly disagree). The total score is the mean of item scores and hence can range from 1 to 4. A higher score indicates greater self-efficacy. In this study sample, Cronbach's *α* for the scale was .74 indicating good internal reliability.

**Depressive symptoms** was assessed with the 16-item version of Center for Epidemiologic Studies Depression Scale (Hautzinger, 1988; Radloff, 1977, p. 237). A sample item is “*During the past week, I felt depressed…”* The full list of items is reported in Supplemental Table 1. Each item is answered on a scale from 1 to 4. The total score is the sum of all items (range: 0–48). High values indicate more depressive symptoms. Cronbach's alpha for this scale was 0.77 indicating good internal reliability.

**Satisfaction with life** was measured with the five-item Satisfaction With Life scale (Diener et al., 1985). Example questions are “*In most ways my life is close to my ideal”* and “*The conditions of my life are excellent.”* Each item can be answered on a scale from 1 to 5 (1 = Strongly agree; 2 = Agree; 3 = Neither agree nor disagree; 4 = Disagree; 5 = Strongly disagree). The total score is the mean of item scores, with a higher score indicating greater satisfaction with life. In this study's sample, Cronbach's *α* for the scale was .84 indicating excellent internal reliability.

**Sociodemographic variables** comprised age, sex, education (low; medium; sophisticate; high), and marital status (married and living together; married but living separated; divorced; widowed).

**Hours of care per week** were assessed with the single-item question: “*How much time do you spend per week helping the person you support?.”* This was used as a continuous variable in the analyses.

**Relationship to the person with dementia** was a categorical variable including the following options: 1 = partner spouse of the person with dementia; 2 = children of the person with dementia; 3 = other type of relationship to the person with dementia.

**Number of health conditions** consisted in the count of the following chronic conditions: high cholesterol; diabetes or high blood sugar levels; high blood pressure; heart attack, angina pectoris; cardiac insufficiency including coronary art; stroke; circulatory disorders in the brain; circulatory disorders in the legs; joint degeneration (arthrosis) of the hips; osteoporosis; inflammatory joint or spinal disease / arthritis; chronic pulmonary disease; cancer, malignant tumor including leukemia; stomach ulcer, intestinal ulcer; incontinence; mental illness (e.g., panic attacks, depression); Parkinson disease; glaucoma or macular degeneration; other chronic disease or health condition.

### Analyses

Summary statistics for study variables for both dementia caregivers and non-caregivers were reported. Linear regression models were used to investigate whether the selected self-perceptions of aging measure (one of felt age, attitudes towards own aging, and AgeCog scales), caregiving status, and their interaction explains a significant amount of variance in each of self-efficacy, depressive symptoms, and satisfaction with life. For those interactions that results significant, stratified analyses for caregivers and non-caregivers were conducted for the given self-perceptions of aging measures and the given caregiving correlate (i.e., self-efficacy, depressive symptoms, and satisfaction with life). For all regression models, simple and multivariate models adjusting for age, sex, education, marital status, and number of chronic health conditions were estimated. For caregivers, an additional third model was estimated controlling for hours of caregiving for week and relationship to the person with dementia, in addition to age, sex, education, marital status, and number of chronic health conditions. For all models unstandardized (B) and standardized (ß) regression coefficients were reported. The effect size was indicated by standardized regression coefficients. Coefficients  ≤ 0.09 were considered negligible; between 0.10 and 0.29 were considered small, between 0.30 and 0.49 were considered moderate, and  ≥ 0.50 were considered large (Cohen, 1988). Analyses were conducted using STATA version 17 (StataCorp, 2025).

## Results

### Descriptive Statistics

Participants comprised 190 dementia caregivers and 4480 non-caregivers (Table 1) residing in Germany. Mean age for dementia caregivers was 65.69 years (range 48 to 89 years) whereas mean age for non-caregivers was 68.81 years (range: 46–98 years). Among dementia caregivers 65.4% were women whereas among non-caregivers 49.4% were women. In both subsamples about 40% of participants achieved high education, most participants were married and lived with their spouse. Both dementia caregivers and non-caregivers on average felt 10% younger than their age and had a mix of positive and negative attitudes towards own aging. Among AgeCog scales, both dementia caregivers and non-caregivers scored the highest in the continuous growth and ongoing development scale, followed in decreasing order by the physical losses scale and the social losses scale. Non-caregivers reported slightly higher social losses than dementia caregivers. Overall, results on self-perceptions of aging indicators suggest participants reported a mix of positive and negative self-perceptions of aging. Both caregivers and non-caregivers reported few depressive symptoms, high self-efficacy, and high levels of satisfaction with life. On average, dementia caregivers provided 18.48 h of care to the person with dementia per week. About 22% of dementia caregivers were providing care to a spouse; about 39% were providing care to a parent; and 38% were providing care to others.

**Table 1.** Descriptive Statistics of Study Variables for Dementia Caregivers and Non-Caregivers.

	Dementia caregivers		
Variables	Statistics	Non-caregivers	*p*-value
N =	190	4480	
Age, M (SD; range)	65.69 (10.11; 48–89)	68.81 (10.49; 46–98)	.001
Sex, n (%)			
Women	100 (65.4)	1797 (49.4)	.001
Men	53 (34.6)	1842 (50.6)	
Missing	37	841	
Education, n (%)			
Low	7 (3.7)	202 (4.5)	.449
Medium	80 (42.1)	2109 (47.1)	
Sophisticate	29 (15.3)	648 (14.5)	
High	74 (39.0)	1521 (40.0)	
Marital status, n (%)			
Married and living together	142 (74.7)	3026 (67.6)	.073
Married and living separated	3 (1.6)	58 (1.3)	
Divorced	17 (9.0)	445 (9.9)	
Widowed	15 (7.9)	684 (15.3)	
Single	13 (6.8)	265 (5.9)	
Missing	0	2	
Hours of caregiving provided per week, M (SD)	18.48 (29.56)		
Relationship to the person with dementia, n (%)			
Spouse	43 (22.6)		
Children	74 (39.0)		
Other	73 (38.4)		
Felt age, M (SD)	0.10 (0.10)	0.10 (0.16)	.912
Missing	2	47	
Attitudes towards own aging, M (SD)	3.01 (0.49)	2.97 (0.56)	.027
Missing	37	853	
Physical losses, M (SD)	10.76 (2.16)	10.95 (2.21)	.298
Missing	17	895	
Social losses, M (SD)	6.75 (1.89)	7.27 (2.23)	.005
Missing	18	900	
Continuous growth and ongoing development, M (SD)	11.78 (2.22)	11.64 (2.24)	.462
Missing	19	890	
Self-efficacy, M (SD)	3.05 (0.41)	3.06 (0.43)	.736
Missing	5	835	
Depressive symptoms, M (SD)	7.65 (6.52)	6.36 (5.96)	.078
Missing	0	3	
Satisfaction with life, M (SD)	3.87 (0.69)	3.91 (0.68)	.414
Missing	18	853	
Number of diagnosed health conditions, M (SD)	2.78 (2.10)	2.83 (2.079	.850
Missing	19	875	

### Self-Efficacy

The interaction between felt age and caregiving status explained a significant amount of variance in self-efficacy at cross-sectional level, suggesting that the association of felt age with self-efficacy differs between dementia caregivers and non-caregivers (Table 2). In the regression model adjusted for age, sex, education, marital status, and number of health conditions, a younger felt age to a greater extent was significantly associated with greater self-efficacy both among dementia caregivers (B = 1.10; 95% CI: 0.37, 1.82; ß = 0.24; R^2^ = 5%) and among non-caregivers (B = 0.17; 95% CI: 0.09, 0.25; ß = 0.07; R^2^ = 0.04%) but the association was stronger among dementia caregivers (Supplemental Tables 2 and 3). In the adjusted regression model, more positive attitudes towards own aging was significantly associated with greater self-efficacy in the overall study sample (B = 0.41; 95% CI: 0.39, 0.43; ß = 0.53), and there were no differences in the association of attitudes towards own aging with self-efficacy between dementia caregivers and non-caregivers. In the adjusted regression model, more perceived physical losses were significantly associated with lower self-efficacy in the overall study sample (B = −0.07; 95% CI: −0.07, −0.06; ß = −0.35), and there were no differences in the association of perceived physical losses and self-efficacy between dementia caregivers and non-caregivers. In the adjusted regression model, more perceived social losses were significantly associated with lower self-efficacy in the overall study sample (B = −0.08; 95% CI: −0.08, −0.07; ß = −0.41), and there were no differences in the association of perceived social losses with self-efficacy between dementia caregivers and non-caregivers. In the adjusted regression model, more continuous growth and ongoing development were associated with greater self-efficacy in the overall study sample (B = 0.11; 95% CI: 0.10. 0.12; ß = 0.58), and there were no differences in the association of perceived continuous growth and ongoing development with self-efficacy between dementia caregivers and non-caregivers.

**Table 2.** Interactions Between Felt Age, Attitudes Towards Own Aging, Physical Losses, Social Losses, and Continuous Growth and Ongoing Development and Caregiving Status as Cross-Sectional Predictors of Self-Efficacy.

Interaction Between Felt Age and Caregiving Status as Cross-Sectional Predictor of Self-Efficacy						
	Unadjusted model			Multiple regression		
Predictors	B (95% CI)	ß	*p*-value	B (95% CI)	ß	*p*-value
Felt age	0.25 (0.16; 0.33)	0.09	<.001	0.17 (0.09; 0.25)	0.07	<.001
Caregiving status	−0.11 (−0.21; −0.003)	−0.05	.042	−0.10 (−0.21; −0.003)	−0.05	.044
Felt age×caregiving status	0.96 (0.21; 1.71)	0.06	.012	0.84 (0.10; 1.58)	0.05	.026

Interaction Between Attitudes Towards Own Aging and Caregiving Status as Cross-sectional Predictor of Self-Efficacy						
	Univariable regression			Multiple regression		
Predictors	B (95% CI)	ß	*p*-value	B (95% CI)	ß	*p*-value
Attitudes towards own aging	0.42 (0.40; 0.44)	0.54	<.001	0.41 (0.39; 0.43)	0.53	<.001
Caregiving status	−0.07 (−0.44; 0.29)	−0.03	.687	−0.04 (−0.41; 0.32)	−0.02	.821
Attitudes towards own aging×caregiving status	0.01 (−0.10; 0.13)	0.02	.807	0.01 (−0.11; 0.13)	0.01	.929

Interaction Between Physical Losses and Caregiving Status as Cross-Sectional Predictor of Self-Efficacy						
	Univariable regression			Multiple regression		
Predictors	B (95% CI)	ß	*p*-value	B (95% CI)	ß	*p*-value
Physical losses	−0.07 (−0.08; −0.07)	−0.39	<.001	−0.07 (−0.07; −0.06)	−0.35	<.001
Caregiving status	−0.28 (−0.61; 0.05)	−0.13	.092	−0.25 (−0.58; 0.07)	−0.12	.129
Physical losses×caregiving status	0.02 (−0.01; 0.05)	0.12	.116	0.02 (−0.01; 0.05)	0.11	.168

Interaction Between Social Losses and Caregiving Status as Cross-Sectional Predictor of Self-Efficacy						
	Univariable regression			Multiple regression		
Predictors	B (95% CI)	ß	*p*-value	B (95% CI)	ß	*p*-value
Social losses	−0.08 (−0.09; −0.08)	−0.44	<.001	−0.08 (−0.08; −0.07)	−0.41	<.001
Caregiving status	0.02 (−0.21; 0.26)	0.01	.839	0.01 (−0.22; 0.24)	0.004	.938
Social losses×caregiving status	−0.01 (−0.05; 0.02)	−0.04	.447	−0.01 (−0.04; 0.02)	−0.03	.540

Interaction Between Continuous Growth and Ongoing Development and Caregiving Status as Cross-Sectional Predictor of Self-Efficacy						
	Univariable regression			Multiple regression		
Predictors	B (95% CI)	ß	*p*-value	B (95% CI)	ß	*p*-value
Continuous growth and ongoing development	0.11 (0.10; 0.11)	0.58	<.001	0.11 (0.10; 0.12)	0.58	<.001
Caregiving status	0.14 (−0.17; 0.44)	0.06	.377	0.14 (−0.17; 0.44)	0.08	.372
Continuous growth and ongoing development×caregiving status	−0.01 (−0.04; 0.01)	−0.08	.277	−0.01 (−0.04; 0.01)	−0.08	.300

### Depressive Symptoms

The interactions between caregiving status and felt age, attitudes towards own aging, and perceived physical losses explained a significant amount of variance in depressive symptoms at cross-sectional level, suggesting that the associations of felt age, attitudes towards own aging, and perceived physical losses with depressive symptoms vary between caregivers and non-caregivers (Table 3). In the adjusted regression model, a younger felt age was associated with fewer depressive symptoms both among dementia caregivers (B = −20.71; 95% CI: −32.09, −9.40; ß = −0.28; R-squared = 7%) and non-caregivers (B = −3.60; 95% CI: −4.68, −2.53; ß = −0.10; R-squared = 1%) but the association was of smaller size among dementia caregivers (Supplemental Tables 1 and 2). In the adjusted regression model, more positive attitudes towards own aging were associated with fewer depressive symptoms among dementia caregivers (B = −6.99, 95% CI: −9.04, −4.93; ß = −0.51; R-squared = 20%) and non-caregivers (−B = 4.51; 95% CI: −4.83, −4.19; ß = −0.44; R-squared = 16%) and the association was stronger in size among dementia caregivers. In the adjusted model, fewer perceived physical losses were associated with fewer depressive symptoms among dementia caregivers (B = 1.11; 95% CI: 0.64; 1.58; ß = 0.35; R-squared = 11%) and non-caregivers (B = 0.75; 95 CI: 0.67, 0.84; ß = 0.29; R-squared = 7%) but the association was of stronger size among dementia caregivers. In the adjusted regression model, more perceived social losses were associated with more depressive symptoms (B = 0.81; 95% CI: 0.73, 0.89; ß = 0.31) both among caregivers and non-caregivers. In the adjusted regression model, less perceived continuous growth and ongoing development was associated with more depressive symptoms (B = −0.77; 95% CI: −0.85, −0.68; ß = 0.30) both among dementia caregivers and non-caregivers.

**Table 3.** Interactions Between Felt Age, Attitudes Towards Own Aging, Physical Losses, Social Losses, and Continuous Growth and Ongoing Development and Caregiving Status as Cross-Sectional Predictors of Depressive Symptoms.

Interaction Between Felt Age and Caregiving Status as Cross-Sectional Predictor of Depressive Symptoms						
	Unadjusted model			Adjusted model		
Predictors	B (95% CI)	ß	*p*-value	B (95% CI)	ß	*p*-value
Felt age	−6.61 (−7.69; −5.53)	−0.18	<.001	−3.61 (−4.69; −2.53)	−0.10	<.001
Caregiving status	2.42 (1.22; 3.62)	0.08	<.001	3.21 (1.88; 4.53)	0.11	<.001
Felt age×caregiving status	−10.75 (−19.01; −2.49)	−0.05	.011	−17.05 (−26.74; −7.37)	−0.08	.001

Interaction Between Attitudes Towards Own Aging and Caregiving Status as Cross-sectional Predictor of Depressive Symptoms						
	Univariable regression			Multiple regression		
Predictors	B (95% CI)	ß	*p*-value	B (95% CI)	ß	*p*-value
Attitudes towards own aging	−4.91 (−5.21; 4.61)	−0.47	<.001	−4.51 (−4.83; −4.19)	−0.43	<.001
Caregiving status	10.42 (5.29; 15.55)	0.35	<.001	9.45 (4.39; 14.52)	0.32	<.001
Attitudes towards own aging×caregiving status	−2.83 (−4.51; −1.15)	−0.29	.001	−2.63 (−4.29; −0.98)	−0.27	.002

Interaction Between Physical Losses and Caregiving Status as Cross-Sectional Predictor of Depressive Symptoms						
	Univariable regression			Multiple regression		
Predictors	B (95% CI)	ß	*p*-value	B (95% CI)	ß	*p*-value
Physical losses	0.91 (0.83; 0.99)	0.34	<.001	0.75 (0.66; 0.84)	0.28	<.001
Caregiving status	−2.29 (−6.81; 2.23)	−0.08	.320	−3.05 (−7.45; 1.34)	−0.10	.173
Physical losses×caregiving status	0.38 (−0.03; 0.80)	0.14	.067	0.43 (0.03; 0.83)	0.16	.035

Interaction Between Social Losses and Caregiving Status as Cross-Sectional Predictor of Depressive Symptoms						
	Univariable regression			Multiple regression		
Predictors	B (95% CI)	ß	*p*-value	B (95% CI)	ß	*p*-value
Social losses	0.96 (0.88; 1.04)	0.37	<.001	0.81 (0.73; 0.89)	0.31	<.001
Caregiving status	−0.90 (−4.16; 2.35)	−0.03	.586	−0.90 (−4.06; 2.25)	−0.03	.574
Social losses×caregiving status	0.47 (0.01; 0.93)	0.11	.047	0.41 (−0.04; 0.86)	0.10	.073

Interaction Between Continuous Growth and Ongoing Development and Caregiving Status as Cross-Sectional Predictor of Depressive Symptoms						
	Univariable regression			Multiple regression		
Predictors	B (95% CI)	ß	*p*-value	B (95% CI)	ß	*p*-value
Continuous growth and ongoing development	−0.87 (−0.95; −0.80)	−0.34	<.001	−0.77 (−0.85; −0.68)	−0.30	<.001
Caregiving status	3.32 (−1.41; 8.06)	0.11	.169	3.82 (−0.76; 8.40)	0.13	.102
Continuous growth and ongoing development×caregiving status	−0.13 (−0.52; 0.27)	−0.05	.534	−0.20 (−0.58; 0.18)	−0.08	.306

### Satisfaction With Life

The interactions of felt age and attitudes towards own aging with caregiving status explained a significant amount of variance in satisfaction with life at cross-sectional level, suggesting that the associations of felt age and attitudes towards own aging with satisfaction with life differ in size between dementia caregivers and non-caregivers (Table 4). In the adjusted regression model, a younger felt age was associated with greater satisfaction with life among dementia caregivers (B = 1.65; 95% CI: 0.46, 2.85; ß = 0.22; R^2^ = 4%), as well as among non-caregivers (B = 0.27; 95% CI: 0.15, 0.40; ß = 0.07; R^2^ = 1%), but the association was stronger in size among dementia caregivers (Supplemental Tables 1 and 2). In the adjusted regression model, more positive attitudes towards own aging were associated with greater satisfaction with life among dementia caregiver (B = 0.88; 95% CI: 0.68, 1.08; ß = 0.63; R^2^ = 31%) and non-caregivers (B = 0.76, 95% CI: 0.73, 0.79; ß = 0.63; R^2^ = 33%), but the association was stronger for dementia caregivers.

**Table 4.** Interactions Between Felt Age, Attitudes Towards Own Aging, Physical Losses, Social Losses, and Continuous Growth and Ongoing Development and Caregiving Status as Cross-Sectional Predictors of Satisfaction With Life.

Interaction Between Felt Age and Caregiving Status as Cross-Sectional Predictor of Satisfaction With Life						
	Unadjusted model			Adjusted model		
Predictors	B (95% CI)	ß	*p*-value	B (95% CI)	ß	*p*-value
Felt age	0.44 (0.31; 0.57)	0.11	<.001	0.27 (0.15; 0.40)	0.07	<.001
Caregiving status	−0.21 (−0.37; −0.05)	−0.06	.010	−0.21 (−0.37; −0.06)	−0.06	.007
Felt age×caregiving status	1.64 (0.47; 2.82)	0.07	.006	1.40 (0.27; 2.54)	0.06	.015

Interaction Between Attitudes Towards Own Aging and Caregiving Status as Cross-sectional Predictor of Satisfaction With Life						
	Univariable regression			Multiple regression		
Predictors	B (95% CI)	ß	*p*-value	B (95% CI)	ß	*p*-value
Attitudes towards own aging	0.74 (0.71; 0.77)	0.61	<.001	0.76 (0.72; 0.79)	0.62	<.001
Caregiving status	−0.46 (−1.00; 0.07)	−0.13	.091	−0.60 (1.13; −0.08)	−0.18	.023
Attitudes towards own aging×caregiving status	0.13 (−0.05; 0.30)	0.11	.153	0.17 (0.004; 0.34)	0.16	.045

Interaction Between AgeCog Physical Losses and Caregiving Status as Cross-Sectional Predictor of Satisfaction With Life						
	Univariable regression			Multiple regression		
Predictors	B (95% CI)	ß	*p*-value	B (95% CI)	ß	*p*-value
Physical losses	−0.12 (−0.13; −0.11)	−0.38	<.001	−0.10 (−0.11; −0.09)	−0.34	<.001
Caregiving status	−0.30 (−0.81; 0.22)	−0.09	.263	−0.23 (−0.74; 0.27)	−0.07	.363
Physical losses×caregiving status	0.02 (−0.03; 0.07)	0.07	.375	0.01 (−0.03; 0.06)	0.04	.554

Interaction Between Social Losses and Caregiving Status as Cross-Sectional Predictor of Satisfaction With Life						
	Univariable regression			Multiple regression		
Predictors	B (95% CI)	ß	*p*-value	B (95% CI)	ß	*p*-value
Social losses	−0.14 (−0.15; −0.13)	−0.46	<.001	−0.13 (−0.14; −0.12)	−0.42	<.001
Caregiving status	0.01 (−0.35; 0.38)	0.004	.944	0.03 (−0.33; 0.38)	0.01	.882
Social losses×caregiving status	−0.02 (−0.07; 0.03)	−0.04	.344	−0.02 (−0.07; 0.03)	−0.05	.373

Interaction Between Continuous Growth and Ongoing Development and Caregiving Status as Cross-Sectional Predictor of Satisfaction With Life						
	Univariable regression			Multiple regression		
Predictors	B (95% CI)	ß	*p*-value	B (95% CI)	ß	*p*-value
Continuous growth and ongoing development	0.14 (0.13; 0.15)	0.48	<.001	0.15 (0.14; 0.16)	0.49	<.001
Caregiving status	−0.19 (−0.71; 0.32)	−0.06	.463	−0.28 (−0.77; 0.22)	−0.08	.271
Continuous growth and ongoing development×caregiving status	0.01 (−0.03; 0.05)	0.04	.621	0.02 (−0.02, 0.06)	0.06	.399

For both dementia caregivers and non-caregivers, greater perceived physical losses were associated with less satisfaction with life (B = −0.10; 95% CI: −0.11, −0.09; ß = −0.34), greater perceived social losses were associated with less satisfaction with life (B = −0.08; 95% CI: −0.08, −0.07; ß = −0.42), greater perceived continuous growth and ongoing development was associated with greater satisfaction with life (B = 0.15; 95% CI: 0.14, 0.16; ß = 0.49).

## Discussion

Using a sample of middle-aged and older individuals, this study estimated the cross-sectional associations of self-perceptions of aging indicators (felt age; attitudes towards own aging; perceived physical losses; perceived social losses; and continuous growth and ongoing development) with self-efficacy, depressive symptoms, and satisfaction with life in dementia caregivers and non-caregivers. It is found that a younger felt age, more positive attitudes towards own aging, fewer perceived physical losses, fewer perceived social losses, and more continuous growth and ongoing development were cross-sectionally associated with greater self-efficacy, fewer depressive symptoms, and greater satisfaction with life in the overall study sample comprising caregivers and non-caregivers. The current study results are aligned with previous evidence reporting that a younger felt age was found cross-sectionally associated with greater self-efficacy, fewer depressive symptoms, and higher satisfaction with life also in a UK cohort of more than 1000 dementia caregivers (Sabatini et al., 2024a, 2024b). The current study results also extend previous evidence to other self-perceptions of aging indicators, namely attitudes towards own aging and the AgeCog scales.

The current study also investigated whether the size of the above associations differ between dementia caregivers and non-caregivers. The study found that the following associations were all stronger in size among dementia caregivers compared to non-caregivers: a younger felt age with greater self-efficacy; a younger felt age more positive attitudes towards own aging; fewer perceived physical losses with fewer depressive symptoms; and a younger felt age and more positive attitudes towards own aging with greater satisfaction with life. Hence, study results suggest that, not only are self-perceptions of aging cross-sectionally associated with self-efficacy, depressive symptoms, and satisfaction with life in dementia caregivers, but dementia caregiving may have a role in the associations between self-perceptions of aging and outcomes such as self-efficacy, depressive symptoms, and satisfaction with life. In fact, it is possible that self-perceptions of aging may be more salient for dementia caregivers than non-caregivers, at least in terms of the constructs we measured in this study.

Theoretical models of awareness of aging hypothesize that life events may shape individuals self-perceptions of aging (Diehl & Wahl, 2024); our results suggest that life events such as being a caregiver for a person with dementia may also influence the strength of the associations between self-perceptions of aging and their correlates. However, future studies should focus on identifying what it is about caregiving that influences these associations. It may be that positive self-perceptions of aging is a psychological resource that becomes particularly useful for maintenance of self-efficacy, good mood, and high satisfaction with life when facing age-related challenges and difficult roles such as caregiving for an old person with dementia. Indeed, those who feel good about themselves and their own aging may increase their self-efficacy as a result of being able to provide instrumental and emotional support to another person (Gallagher et al., 2011). At the same time, negative self-perceptions of aging may exacerbate caregivers’ risk of experiencing a decline in self-efficacy, depressive symptoms, and low satisfaction with life.

Several psychological theories provide explanation for the association of more negative self-perceptions of aging with poorer mood. For example, according to Terror Management Theory (Greenberg et al., 1986; Pyszczynski et al., 2015) those with more negative self-perceptions of aging are more likely to experience depressive symptoms because of their increased awareness of age-related health conditions and mortality. It may be that among those with more negative self-perceptions of aging dementia caregiving increases thoughts related to one's susceptibility to illnesses and mortality and, consequently, depressive symptoms. Next, previous evidence and Hopelessness Theory of Depression (Abramson et al., 1989; Tully-Wilson et al., 2021) suggest that people with negative self-perceptions of aging are more likely to feel they have little to no control over their aging and their health and, because of this, they are more likely to experience depressive symptoms. Again, it may be that caregiving for a person with dementia heightens feelings of hopelessness as many dementia caregivers and middle-aged and older people in general erroneously think they will also develop dementia (Andrews et al., 2017; Pacifico et al., 2022). Finally, according to Contact Theory it may be that simply being in contact with a person with dementia who, in addition to facing significant cognitive and functional limitations, may experience negative changes in how they perceive themselves and a decrease in their mood, caregivers also experience both more negative self-perceptions and poorer mood (Borenstein-Laurie et al., 2023).

Many dementia caregivers at some point feel not able to deal with dementia specific symptoms and behaviors, and experience some sort of depressive symptoms (Joling et al., 2010; Mahoney et al., 2005; Wulff et al., 2020). Because of this many interventions have been designed with the aim to support caregivers’ self-efficacy and mental health (Hopkinson et al., 2019; Tang & Chan, 2016). The current study findings about the connections between dementia caregivers’ self-perceptions of aging and their self-efficacy, depressive symptoms, and satisfaction with life offer insight about psychological components that can be incorporated into caregivers’ interventions (Joling et al., 2010; Mahoney et al., 2005; Wulff et al., 2020). Indeed, self-perceptions of aging are malleable and subject to change through psychoeducational and behavioral interventions (Gilhooly et al., 2016; Knight et al., 2021) that have been showed effective, not only with regard to the promotion of more positive self-perceptions of aging, but also with regard to the promotion of greater mental health and emotional wellbeing.

This study has several limitations. A first limitation of this study is its cross-sectional nature, which did not allow to test for bidirectional associations between self-perceptions of aging indicators of caregiving outcomes. Hence, it could be possible that self-efficacy, depressive symptoms, and satisfaction with life predict self-perceptions of aging. Although the German Ageing Survey is a longitudinal cohort study that started in 1996, a question asking participants whether they care for someone with dementia has only been included in 2021. In the previous assessment points, participants were simply asked whether they cared for someone, but were not asked to specify the condition of the person they were caring for. Longitudinal studies are needed to text the long-term effects of self-perceptions of aging on caregiving outcomes. A second limitation of this study is that only 190 participants were dementia caregivers. This sample size prevented stratified analyses by age and by relationship to the person with dementia (e.g., spousal caregivers vs. children caregivers). Moreover, the number of non-caregivers was substantially higher compared to the number of caregivers and this may have led to more stable results for non-caregivers compared to dementia caregivers. A third limitation of this study is that the dataset included no information on the type of dementia of the person caregivers provide care for. The dataset also did not include any information on how many months/years caregivers have been caring for the person with dementia. Hence, we could not control for these important variables in the analyses.

This study has several strengths. First, a strength of this study is the inclusion of multiple measures of self-perceptions of aging. This is important as different self-perceptions of aging measures often show different results. Importantly, one of the self-perceptions of aging measures included in this study (the AgeCog scales) is multidimensional and captures both positive and negative age-related experiences. This is important as each stage of life involves both gains and losses. This is also one of the first few studies investigating self-perceptions of aging in dementia caregivers, and the first to investigate this in Germany.

To conclude, more positive self-perceptions of aging may be important for the experience of greater self-efficacy, good mood, and satisfaction with life in the general population and especially among dementia caregivers. There is the need of longitudinal data establishing the causality of the associations between self-perceptions of aging with self-efficacy, good mood, and satisfaction with life among dementia caregivers as this may provide essential evidence for the inclusion of components promoting more positive self-perceptions of aging in existing caregiving interventions.

## Supplemental Material

sj-docx-1-ahd-10.1177_00914150251382810 - Supplemental material for Cross-sectional Associations of Self-Perceptions of Aging With Self-Efficacy, Depressive Symptoms, and Satisfaction With Life in Dementia Caregivers and Non-Caregivers Supplemental material, sj-docx-1-ahd-10.1177_00914150251382810 for Cross-sectional Associations of Self-Perceptions of Aging With Self-Efficacy, Depressive Symptoms, and Satisfaction With Life in Dementia Caregivers and Non-Caregivers by Serena Sabatini and Shelbie Turner in The International Journal of Aging and Human Development
