# Supplementary material for: Cross-sectional Associations of Self-Perceptions of Aging With Self-Efficacy, Depressive Symptoms, and Satisfaction With Life in Dementia Caregivers and Non-Caregivers
Source: Int J Aging Hum Dev. 2025 Oct 9;102(4):459–81. doi: 10.1177/00914150251382810 (PMC13068312; doi:10.1177/00914150251382810)
Supplement: sj-docx-1-ahd-10.1177_00914150251382810 - Supplemental material for Cross-sectional Associations of Self-Perceptions of Aging With Self-Efficacy, Depressive Symptoms, and Satisfaction With Life in Dementia Caregivers and Non-Caregivers [file sj-docx-1-ahd-10.1177_00914150251382810.docx]

| **Supplementary Table 1.**  Items included in the German version of the CESD. |
| --- |
| Item |
| I was bothered about things that usually don’t bother me |
| 2. I felt that I could not shake off the blues even with the help from my friends / family who tried to cheer me up |
| 3. I struggled to concentrate myself |
| 4. I felt depressed |
| 5. I felt that everything I did was an effort |
| 6. I thought my life had been a complete failure |
| 7. I was scared |
| 8. My sleep was restless |
| 9. I was in a happy mood* |
| 10. I talked less than usual |
| 11. I felt lonely |
| 12. I enjoyed life* |
| 13. I felt sad |
| 14. I felt that people dislike me |
| 15. I couldn't get myself to do anything |
| 16. I thought of the future with hope |
| ***Notes.*** Reverse-coded items. |

| **Supplementary Table 2.**  Cross-Sectional Associations of Felt Age With Self-Efficacy, Depressive Symptoms, and Satisfaction With Life in Dementia Caregivers | | | | | | | | | | | | | | |
| --- | --- | --- | --- | --- | --- | --- | --- | --- | --- | --- | --- | --- | --- | --- |
| **Association of Felt Age With Self-Efficacy** | | | | | | | | | | | | | | |
|  | Model 1 | | | | Model 2 | | | | | Model 3 | | | | |
| Predictors | B (95% CI) | ß | p-value | R^2^ | B (95% CI) | ß | | p-value | R^2^ | B (95% CI) | ß | p-value | | R^2^ |
| Felt age | 1.21 (0.51; 1.90) | 0.27 | .001 | 7% | 1.10 (0.37, 1.82) | 0.24 | | .003 | 5% | 0.95 (0.64; 1.26) | 0.23 | .001 | | 5% |
| **Association of Felt Age With Depressive Symptoms** | | | | | | | | | | | | | | |
|  | Model 1 | | | | Model 2 | | | | | Model 3 | | | | |
| Predictors | B (95% CI) | ß | p-value | R^2^ | B (95% CI) | ß | p-value | | R^2^ | B (95% CI) | ß | p-value | | R^2^ |
| Felt age | -17.36 (-26.23; -8.50) | -0.27 | <.001 | 7% | -20.71 (-32.09; -9.40) | -0.28 | <.001 | | 7% | -12.64 (-16.89; -8.39) | -0.21 | <.001 | | 4% |
| **Association of Attitudes Towards Own Aging With Depressive Symptoms** | | | | | | | | | | | | | | |
|  | Model 1 | | | | Model 2 | | | | | Model 3 | | | | |
| Predictors | B (95% CI) | ß | p-value | R^2^ | B (95% CI) | ß | p-value | | R^2^ | B (95% CI) | ß | p-value | | R^2^ |
| Attitudes Towards Own Aging | -7.74 (-9.59; -5.89) | -0.56 | <.001 | 31% | -6.99 (-9.04; -4.93) | -0.51 | <.001 | | 0.20 | -4.80 (-5.61; -4.00) | -0.41 | .001 | | 14% |
| **Association of Physical Losses With Depressive Symptoms** | | | | | | | | | | | | | | |
|  | Model 1 | | | | Model 2 | | | | | Model 3 | | | | |
| Predictors | B (95% CI) | ß | p-value | R^2^ | B (95% CI) | ß | p-value | | R^2^ | B (95% CI) | ß | p-value | | R^2^ |
| Physical losses | 0.92 (0.85; 0.99) | 0.35 | <.001 | 12% | 1.11 (0.64; 1.58) | 0.35 | <.001 | | 11% | 0.83 (0.63; 1.02) | 0.30 | <.001 | | 8% |
| **Association of Felt Age With Satisfaction With Life** | | | | | | | | | | | | | | |
|  | Model 1 | | | | Model 2 | | | | | Model 3 | | | | |
| Predictors | B (95% CI) | ß | p-value | R^2^ | B (95% CI) | ß | p-value | | R^2^ | B (95% CI) | ß | p-value | R^2^ | |
| Felt age | 2.09 (0.91; 3.26) | 0.28 | .001 | 8% | 1.65 (0.46; 2.85) | 0.22 | .007 | | 4% | 0.87 (0.37; 1.37) | 0.13 | .001 | 2% | |
| **Association of Attitudes Towards Own Aging With Satisfaction With Life** | | | | | | | | | | | | | | |
|  | Model 1 | | | | Model 2 | | | | | Model 3 | | | | |
| Predictors | B (95% CI) | ß | p-value | R^2^ | B (95% CI) | ß | p-value | | R^2^ | B (95% CI) | ß | p-value | R^2^ | |
| Attitudes towards own aging | 0.87 (0.69; 1.05) | 0.62 | <.001 | 39% | 0.88 (0.68; 1.08) | 0.63 | <.001 | | 31% | 0.72 (0.63; 0.81) | 0.54 | <.001 | 25% | |
| ***Notes.*** Model 1: unadjusted model. Model 2: adjusted for adjusted for age, sex, education, marital status, number of health conditions. Model 3: adjusted for age, sex, education, marital status, number of health conditions, hours of care provided to the person with dementia per week, and relationship to the person with dementia. N= 190. Results for covariates are available upon request to the first author. | | | | | | | | | | | | | | |

| **Supplementary Table 3.**  Cross-Sectional Associations of Felt Age With Self-Efficacy, Depressive Symptoms, and Satisfaction With Life in Non-Caregivers | | | | | | | | | | | | |
| --- | --- | --- | --- | --- | --- | --- | --- | --- | --- | --- | --- | --- |
| **Association of Felt Age With Self-Efficacy** | | | | | | | | | | | | |
|  | Univariable regression | | | | | | | | Multiple regression | | | |
| Predictors | B (95% CI) | ß | p-value | | R^2^ | | | | B (95% CI) | ß | p-value | R^2^ |
| Felt age | 0.25 (0.16; 0.33) | 0.10 | <.001 | | 1% | | | | 0.17 (0.09; 0.25) | 0.07 | <.001 | 0.4% |
| **Association of Felt Age With Depressive Symptoms** | | | | | | | | | | | | |
|  | Univariable regression | | | | | | | | Multiple regression | | | |
| Predictors | B (95% CI) | ß | | p-value | | | R^2^ | | B (95% CI) | ß | p-value | R^2^ |
| Felt age | -6.61 (-7.69; -5.54) | -0.18 | | <.001 | | | 3% | | -3.60 (-4.68; -2.53) | -0.10 | <.001 | 1% |
| **Association of Attitudes Towards Own Aging With Depressive Symptoms** | | | | | | | | | | | | |
|  | Univariable regression | | | | | | | | Multiple regression | | | |
| Predictors | B (95% CI) | ß | | p-value | | | R^2^ | | B (95% CI) | ß | p-value | R^2^ |
| Attitudes towards own aging | -4.91 (-5.20; -4.61) | -0.48 | | <.001 | | | 23% | | -4.51 (-4.83; -4.19) | -0.44 | <.001 | 16% |
| **Association of Physical Losses With Depressive Symptoms** | | | | | | | | | | | | |
|  | Univariable regression | | | | | | | Multiple regression | | | | |
| Predictors | B (95% CI) | ß | | p-value | | R^2^ | | B (95% CI) | | ß | p-value | R^2^ |
| Physical losses | 0.91 (0.83; 0.99) | 0.35 | | <.001 | | 12 | | 0.75 (0.67; 0.84) | | 0.29 | <.001 | 7% |
| **Association of Felt Age With Satisfaction With Life** | | | | | | | | | | | | |
|  | Univariable regression | | | | | | | Multiple regression | | | | |
| Predictors | B (95% CI) | ß | | p-value | | R^2^ | | B (95% CI) | | ß | p-value | R^2^ |
| Felt age | 0.44 (0.31; 0.57) | 0.11 | | <.001 | | 1% | | 0.27 (0.15; 0.40) | | 0.07 | <.001 | 1% |
| **Association of Attitudes Towards Own Aging With Satisfaction With Life** | | | | | | | | | | | | |
|  | Univariable regression | | | | | | | Multiple regression | | | | |
| Predictors | B (95% CI) | ß | | p-value | | R^2^ | | B (95% CI) | | ß | p-value | R^2^ |
| Attitudes towards own aging | 0.74 (0.71; 0.77) | 0.62 | | <.001 | | 38% | | 0.76 (0.73; 0.79) | | 0.63 | <.001 | 33% |
| ***Notes.*** All multiple regression models are adjusted for age, sex, education, marital status, number of health conditions, hours of care provided to the person with dementia per week, and relationship to the person with dementia. N= 4480. Results for covariates are available upon request to the first author. | | | | | | | | | | | | |
